# Humoral immune response to SARS-CoV-2 in five different groups of individuals at different environmental and professional risk of infection

**DOI:** 10.1038/s41598-021-04279-4

**Published:** 2021-12-30

**Authors:** Silvia Novello, Massimo Terzolo, Berchialla Paola, Martina Gianetta, Valentina Bianco, Francesca Arizio, Dalila Brero, Anna Maria Elena Perini, Adriana Boccuzzi, Valeria Caramello, Alberto Perboni, Fabio Bellavia, Giorgio Vittorio Scagliotti

**Affiliations:** 1 Department of Oncology at San Luigi Hospital, University of Torino, Regione Gonzole 10, 10043 Orbassano, Torino Italy; 2 Department of Clinical & Biological Sciences at San Luigi Hospital, University of Torino, Regione Gonzole 10, 10043 Orbassano, Torino Italy; 3 Emergency Care Division, San Luigi Hospital, Regione Gonzole 10, 10043 Orbassano, Torino Italy; 4 Respiratory Medicine Division, San Luigi Hospital, Regione Gonzole 10, 10043 Orbassano, Torino Italy

**Keywords:** Risk factors, Epidemiology

## Abstract

It is partially unknown whether the immune response to severe acute respiratory syndrome coronavirus 2 (SARS‐CoV‐2) infection persists with time. To address this issue, we detected the presence of SARS-CoV-2 antibodies in different groups of individuals previously diagnosed with COVID-19 disease (group 1 and 2), or potentially exposed to SARS-CoV-2 infection (group 3 and 4), and in a representative group of individuals with limited environmental exposure to the virus due to lockdown restrictions (group 5). The primary outcome was specific anti-SARS-CoV-2 antibodies in the different groups assessed by qualitative and quantitative analysis at baseline, 3 and 6 months follow-up. The seroconversion rate at baseline test was 95% in group 1, 61% in group 2, 40% in group 3, 17% in group 4 and 3% in group 5. Multivariate logistic regression analysis revealed male gender, close COVID-19 contact and presence of COVID-19 related symptoms strongly associated with serological positivity. The percentage of positive individuals as assessed by the qualitative and quantitative tests was superimposable. At the quantitative test, the median level of SARS-CoV-2 antibody levels measured in positive cases retested at 6-months increased significantly from baseline. The study indicates that assessing antibody response to SARS-CoV-2 through qualitative and quantitative testing is a reliable disease surveillance tool.

## Introduction

Severe acute respiratory syndrome coronavirus 2 (SARS‐CoV‐2) is a coronavirus responsible of an acute respiratory disease known as coronavirus disease 2019 (COVID‐19)^1,2^.

At the outbreak of the pandemic, the identification of antigenic structures involved in the immune response and the development of serological diagnostic tests were considered research priorities. Currently, studies of SARS-CoV-2 seroprevalence became progressively relevant to inform public health policies based on the risk of either transmitting or acquiring infection^3^. It is presently unknown, however, if infection with SARS-CoV-2 in humans protects against future re-infection and, if so, for how long. Common human beta-coronaviruses induce neutralizing antibodies that can last for years and provide protection against re-infection or induce an attenuated disease when individuals get re-infected^4^. Following COVID 19 disease checking for the presence of anti-SARS-CoV-2 antibodies and assessing the evolution over time of their levels may provide key knowledge to guide individual and population conduct and safety practices^5^.

A recent systematic review included 45 cross-sectional studies that analyzed antibody response mostly in small groups of subjects with different degrees of disese severity during the first 28 days from onset of disease, while only few studies had a follow-up of more than 3 months. Evidence was rated as moderate that most infected individuals develop IgM and IgG anti-SARS-CoV-2 antibodies, with IgG persisting for at least 4 months, while evidence is low on the persistence of neutralizing antibody activity for several months^6^. Indeed, studies on humoral immunity elicited by SARS-CoV-2 infection generated some debate on its longevity^7^.

The aims of this prospective study were: (1) to assess the presence of SARS-CoV-2 antibodies in different groups of individuals who have been previously diagnosed with COVID-19 disease (group 1 and (2), or potentially exposed to SARS-CoV-2 infection (group 3 and 4), and in a representative group of individuals with limited environmental exposure to the virus (group 5); (3) to assess the the serum persistence over time of SARS-CoV-2 antibodies in individuals with previously confirmed COVID-19 disease.

## Methods

### Study design

The primary outcome of the study was to assess the presence of specific anti-SARS-CoV-2 antibodies in the different study groups at baseline, at 3 months in all individuals and therefore at 6 months in the individuals who tested positive at the initial or first follow-up serological test. The study was approved by the Institutional Review Board of the San Luigi Hospital (Torino, Italy) in late May 2020.

Baseline plasma samples were collected between June 15 and July 31, 2020, when the first wave of epidemic had to a large extent receded. The study was conducted according to the criteria set by the Declaration of Helsinki and each subject signed an informed consent before participating to the study. An online or paper self-administered questionnaire was collected for each participating individual asking for information about SARS-CoV-2 risk exposure, professional role in the hospital, timing of positivity/negativity to nasopharyngeal swab (and, if positive, when a double negative test was obtained), COVID-19 related symptoms and previously diagnosed co-morbidities.

Group 1 included individuals with a confirmed diagnosis of SARS-CoV-2 infection by a positive RT-PCR virus test on nasopharyngeal or oropharyngeal swab. Group 2 comprised individuals with suspected SARS-CoV-2 infection due to suggestive clinical features with a negative RT-PCR virus test on at least two nasopharyngeal or oropharyngeal swabs. At the time of the baseline serological test groups 1 and 2 patients were already discharged from hospital and completely recovered. Group 3 included contact or co-exposure with confirmed cases of SARS-CoV-2 infection (household contacts). Group 4 included individuals working in the hospital setting (healthcare workers who were involved in the patient care either in COVID-19 or non-COVID-19 dedicated wards and other occupations) without a positive history for SARS-CoV-2 infection. Group 5 included individuals without occupational risk, living in a geographic area of SARS-CoV-2 outbreak under lockdown restrictions, who did not test positive to the RT-PCR test. For all the enrolled individuals, peripheral blood samples was collected again at month 3 and those tested positive at first and/or second round were tested again at month 6 for longitudinal monitoring of SARS-CoV-2 immune response.

As of September 2020, a new assay became commercially available (Elecsys Anti-SARS-CoV-2 S) to measure the quantitative level of antibodies against SARS-CoV-2. All baseline blood samples tested positive at the semiquantitative assay, adequately stored at − 80 °C, were retested with this new assay to compare the qualitative information previously reported with the quantitative assessment.

At the 3-month follow up, all the enrolled individuals underwent a second blood test to compare qualitatively baseline results with those at the 3-month follow up and to assess if additional individuals turned up to be positive. At 6-month follow up only individuals positive at baseline and/or at 3-month follow up were tested again with the quantitative test.

Secondary outcome measures include the analysis of factors associated with seroconversion and antibody levels both at baseline and during follow-up evaluations.

### Laboratory investigation

Plasma samples adequately stored at – 80 °C were subsequently analyzed in a central laboratory using the Elecsys® Anti‑SARS‑CoV‑2 test an immunoassay for qualitative in vitro detection of antibodies (pan-Ig: IgM, IgG, IGA) to SARS‑CoV‑2 in human serum and plasma using a cobas e801 analyzer (Roche Diagnostics International Ltd, Rotkreuz, Switzerland).

Measurement of Anti-SARS-CoV-2 was performed following the manufacturer’s instructions^8^.

The test detects antibodies in serum or plasma, collected using standard sampling tubes. The results are reported as numeric values in form of a cut-off index (COI; signal sample/cutoff) as well as in form of a qualitative results non-reactive (COI < 1.0; negative) and reactive (COI ≥ 1.0; positive).

Elecsys® Anti-SARS-CoV-2 S is an immunoassay for the in vitro quantitative determination of antibodies (including IgG) to the SARS-CoV-2 spike (S) protein receptor binding domain (RBD) in human serum and plasma. The linear range of the test is 0.4–250 U/ml. Clinical sensitivity ranges from 88.6% (0–6 days post-PCR confirmation) to 100% (24–36 days) with an analytic specificity of 100% for potential cross-reactive samples, clinical specificity of 100%. The Elecsys^®^ Anti-SARS-CoV-2 S assay was compared to a VSV-based pseudo-neutralization assay in 15 clinical samples from individual patients to assess correlation to serum neutralization capacity^9^.

### Sample handling

Anonymized (two side delinked), frozen, residual samples were thawed to room temperature andhomogenized using a slow rotating system or by inverting slowing five times prior to assaying to avoid the production of foam. Before testing, samples were visually inspected to check that they did not contain clots/precipitates, foam or droplets at the container walls. Assay results were obtained via instrument export files.

All the diagnostic testing process has been performed by an external, independent laboratory (Life Brain Laboratory, Ovada, Italy) completely blinded of clinical data. The diagnostic tests were kindly provided free of charge by Roche Diagnostics, Italy.

### Statistical analysis

Continuous variables were reported as median and interquartile range (IQR). Categorical variables are reported as number and percentage. Kruskal–Wallis, Chi-square test and Fisher's exact tests were used to compare continuous and categorical variables, as appropriate. Mann–Whitney test with Hochberg adjustment was performed for pairwise comparison between exposure factors and antitboldy levels. Intraclass correlation coefficient was computes as agreement measure between the in vitro quantitative and qualitative assay of antibodies.

Multivariable and penalized logistic regression were carried out to test for association between exposure risk factors and (1) antibody levels dichotomized as positive (cutoff index [COI] ≥ 1.0) or negative (COI < 1) response, and (2) positive or negative swab test at baseline. The model selection strategy was based on clinical discussion and statistical automated procedures. The best fitting model was chosen on the basis of the Akaike information criterion and further discussed. Interaction among variables was checked in a similar way. Finally, a multivariate model was built and evaluated using a graphical examination of residual diagnostics. Discrimination Index D (the higher the better) and the Somer concordance index Dxy (the closer to 1 in absolute value the better) were also evaluated.

A multilevel quantile regression was used to analyze the association between risk factors for exposure and quantitative antibody levels measured with the Elecsys® Anti‑SARS‑CoV‑2 S test at baseline and 6-month follow up. Median regression was used. The quantile regression was chosen to avoid data transformation due to the non-normal distribution of the errors and 95% confidence intervals were estimated by bootstrapping (500 samples). Random effects on individual were included to account for the repeated measures. Variance–covariance structure of random effects was modeled assuming uncorrelated and Laplace random effects. Goodness-of-fit was assessed through residuals inspection. Statistical significance was set at 5%. All analyses were performed using R version 4.0.2. Default choices were adopted to set up reference groups in multivariable models.

## Results

The baseline blood samples were collected from 1016 individuals but only 989 were fully eligible for the study because of the incomplete information by the self-administered questionnaire, representing a 97% response rate (supplementary figure S1). Table 1 summarizes the distribution and main characteristics of the 5 considered groups. Median age for the entire study population was 47 years (IQR: 34–58) and women were 59%.

**Table 1:** Main characteristics of the individuals enrolled in the 5 study groups.

GROUP		1	2		3	4	5		Overall	p value
N. individuals		(N = 120)	(N = 89)		(N = 128)	(N = 246)	(N = 406)		(N = 989)	
Male (%)		58 (48)	51 (57)		56 (44)	58 (24)	179 (44)		402 (41)	*P* < *0.01*^*2*^
**Age class-years (%)**										*P* < *0.01*^*2*^
< 50		50 (42)	44 (50)		75 (59)	169 (69)	224 (55)		562 (57)	
50–65		49 (41)	27 (30)		36 (28)	74 (30)	117 (29)		303 (31)	
> 65		21 (17)	18 (20)		17 (13)	3 (1)	64 (16)		123(12)	
**Type of occupation (%)**										*P* < *0.01*^*2*^
Physician		8 (7)	3 (3)		8 (6)	38 (15)	1 (0)		58 (6)	
Manager		8 (7)	7 (8)		17 (13)	6 (2)	38 (9)		76 (8)	
Retired Individual		21 (17)	17 (19)		14 (11)	0 (0)	83 (20)		135 (13)	
Researchers/student		3 (3)	10 (11)		20 (16)	62 (25)	76 (19)		171 (17)	
Nurse		28 (23)	12(14)		5 (4)	70 (29)	2 (1)		117 (12)	
Administrative personnel		33 (28)	19 (21)		40 (31)	29 (12)	134 (33)		255 (26)	
Others		15 (12)	12 (14)		17 (13)	39 (16)	55 (14)		138 (14)	
Not reported		4 (3)	9 (10)		7 (6)	2 (1)	17 (4)		39 (4)	
**Environmental and professional risk factors**										*P* < *0.01*^*2*^
None		38 (32)	41 (46)		8 (6)	68 (28)	278 (69)		433 (44)	
COVID-19 close contact		29 (24)	10 (11)		98 (77)	4 (2)	4 (1)		145 (15)	
Healthcare worker*		26 (22)	9 (11)		2 (1)	106 (43)	12 (3)		155 (15)	
Travel to/from high-risk areas		4 (3)	4 (4)		1 (1)	2 (1)	25 (6)		36 (4)	
Homeless/other		11 (9)	15 (17)		5 (4)	28 (11)	82 (20)		141 (14)	
More than one		12 (10)	10 (11)		14 (11)	38 (15)	5 (1)		79 (8)	

*Healthcare workers with multiple risk factors are included in the category ‘More than one’.

COVID-19 related symptoms and comorbidities are reported in supplementary table S1. In Group 1 and 2 only 4% of patients were without any symptom. Comorbidities were absent in 55% of the individuals across the 5 groups ranging from 62% in group 4 to 46% in group 2. Most commonly reported co-morbidities included systemic hypertension, previous chronic respiratory and cardiovascular diseases.

**Figure 1:**
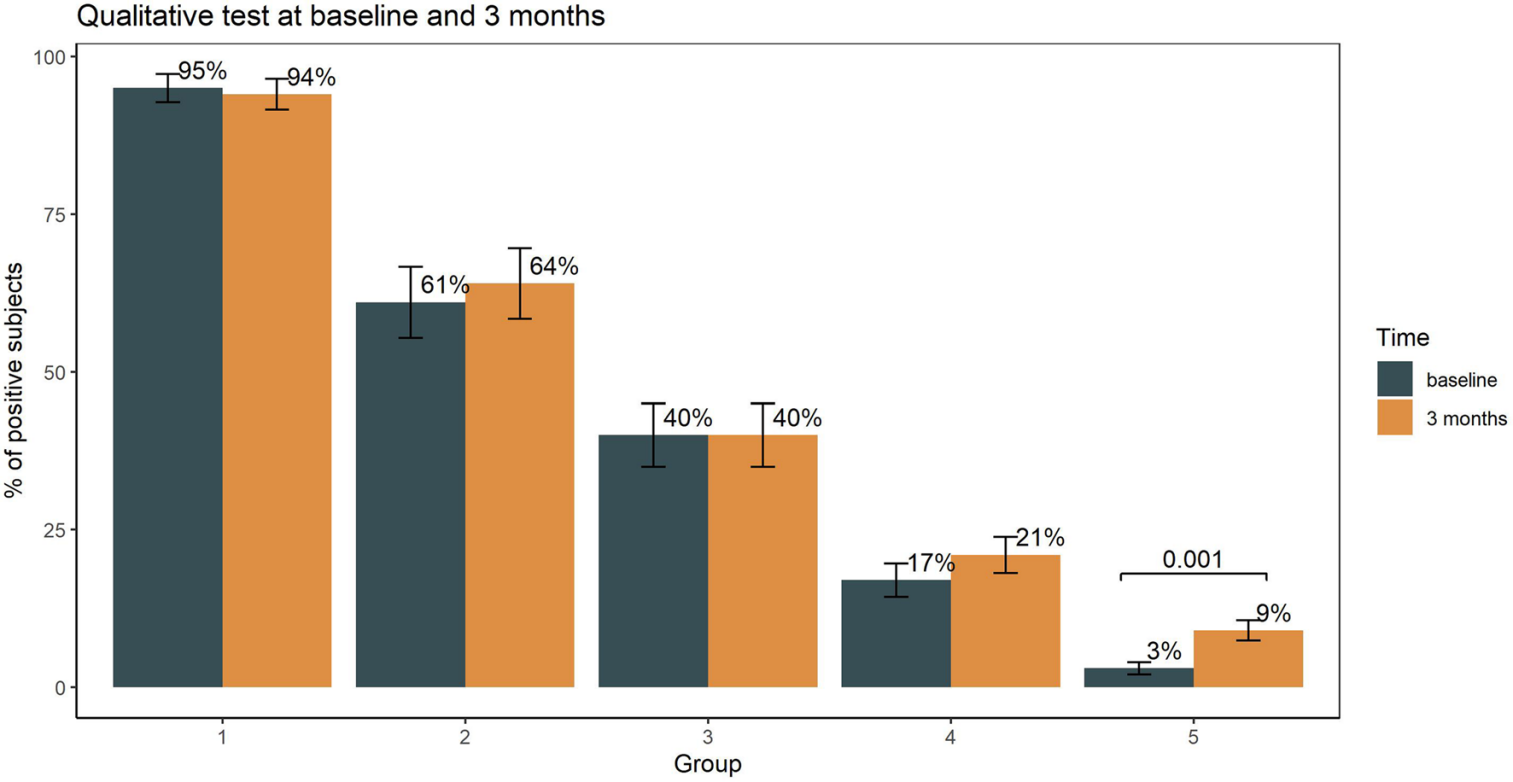
Percentage of subjects who tested positive to qualitative in vitro detection of antibodies at baseline and 3 months follow-up. Statistical differences were examined by McNemar test.

At baseline, seroconversion rate was 95% in group 1, 61% in group 2, 40% in group 3, and 17% in group 4. Among individuals living under lockdown restrictions (group 5), 3% were seropositive. At the 3-month follow up, the percentage of individuals positive at the qualitative anti‑SARS‑CoV‑2 test was almost unchanged in all the groups with the exclusion of group 5 where the positivity rate increased significantly from 3 to 9% (p = 0.001) (Fig. 1).

Overall, there were 474 healthcare workers, ranging from 41 (group 2) to 141 (group 5). Interestingly, the seroconversion rate among healthcare workers in COVID-19 wards (25%) did not significantly differ from that of physicians working in non-COVID-19 wards (26%). Moreover, the serological test yielded positive results in 10% of physicians with a clinical diagnosis of COVID-19 disease based on suggestive clinical features but tested negative on RT-PCR virus test performed within one week from the hospital admission.

**Figure 2:**
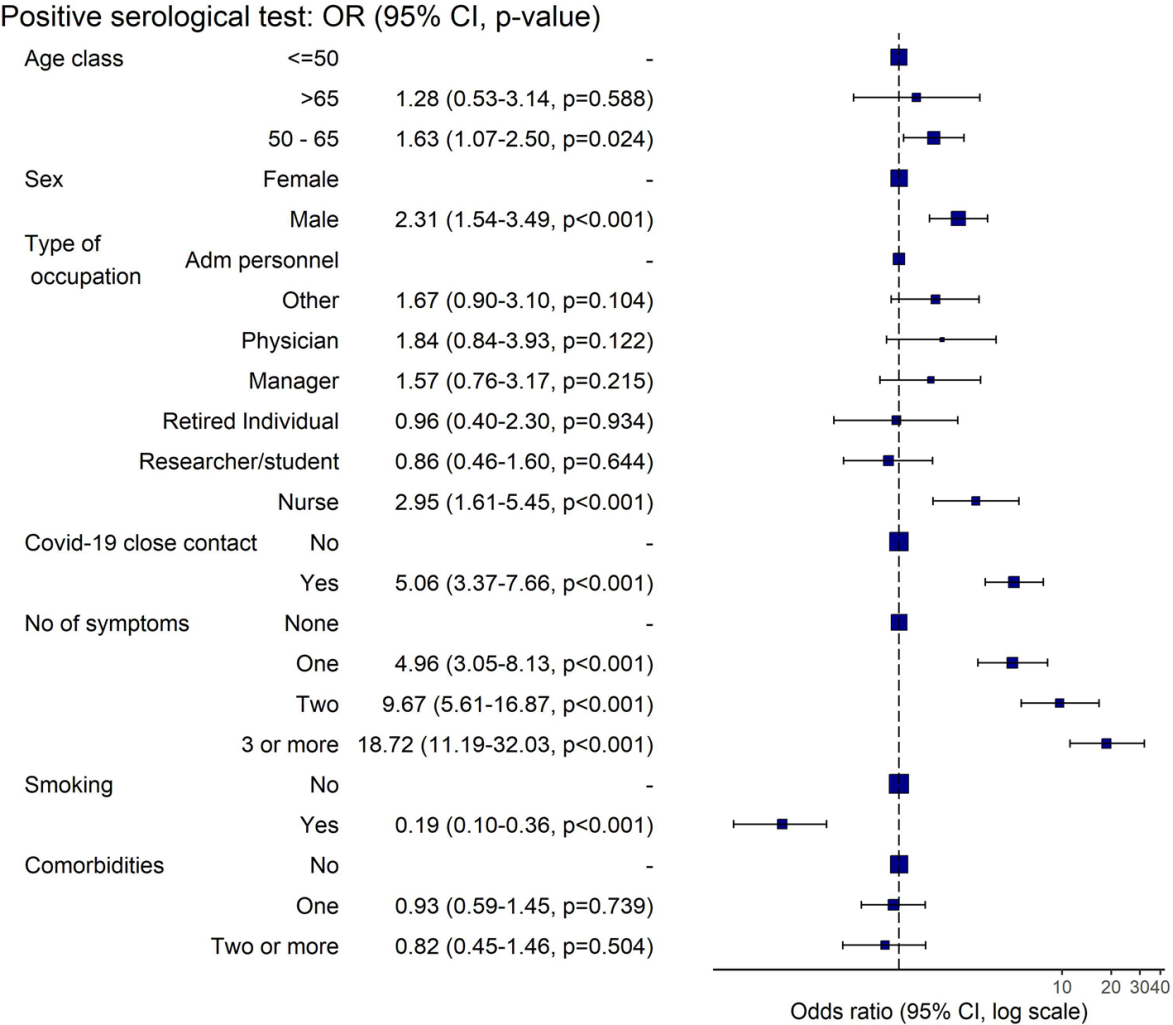
Multivariable logistic regression on qualitative in vitro detection of antibodies levels dichotomized as positive/negative as response at baseline, considering demographic features, exposure to COVID-19 contacts, number of comorbidities, and symptoms. Blue squares and horizontal lines represent ORs and their 95% CI; the dashed vertical line is at OR = 1 (no association). Squares to the left of the dashed line represent protective association, whereas squares to the right are indicative of positive association with Anti‑SARS‑CoV‑2 S test positivity (risk factors). Confidence intervals crossing the dashed vertical line indicate no significant association.

Multivariate logistic regression analysis for the qualititative anti-SARS-CoV-2 test dichotomized as positive or negative and taking into account patients’ and disease characteristics revealed that male gender, close COVID-19 contact and the presence of COVID-19 related symptoms strongly associated with the positivity at the serological test. After adjustment, the risk for positive test was significantly higher in men (OR = 2.31, 95% CI 1.54–3.49, p < 0.001), in individuals aged 50–65 years (OR = 1.63 , 95% CI 1.07–2.50, p = 0.024) and nurse (OR = 2.95, 95% CI 1.61–5–45, p < 0.001). A positive history for tobacco smoking was found to be negatively associated (OR = 0.19, 95% CI 0.10–0.36, p < 0.001). A two-fold increase in risk was found linked to increasing the number of symptoms (Fig. 2).

Two hundred and seventy-two baseline samples (all those tested as positive at the qualitative test and 6 randomly selected samples among those tested negative) were subsequently analyzed with the quantitative test.

Intraclass coefficient measured on qualitative and quantitative determination of antibodies resulted equal to 0.80 (95% CI 0.74; 0.84), showing the percentage of positive individuals at baseline assessed by the qualitative and quantitative tests were superimposable.

**Figure 3:**
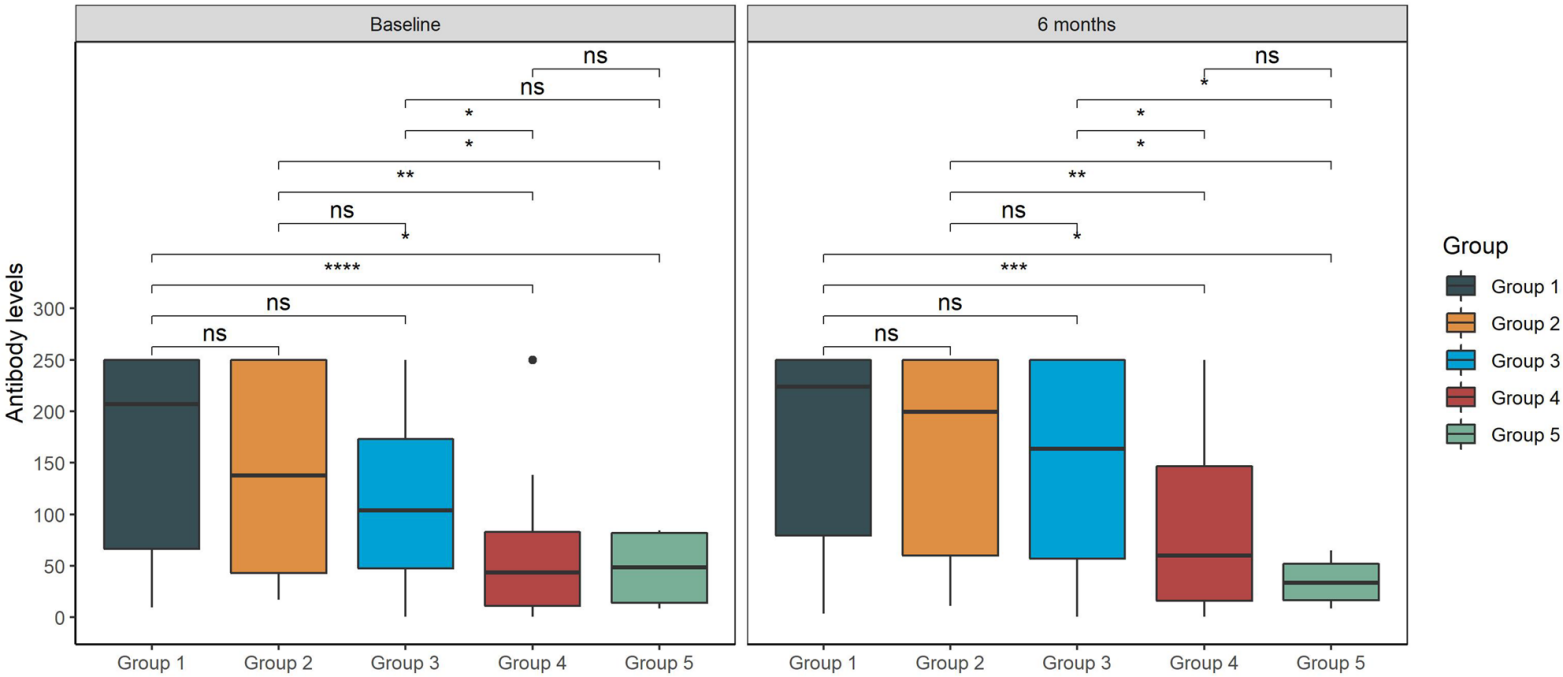
Quantitative test distribution per group stratified according to baseline and 6-month follow up data. Values are expressed in U/ml.

Distribution of antibody levels stratified by group is shown in Fig. 3. Significant higher median antibody levels measured with the Elecsys® Anti‑SARS‑CoV‑2 S test were found in group 1, 2 and 3 compared to group 4 and 5, both at baseline and follow-up test.

A multilevel quantile regression was performed on quantitative antibody levels measured with the Elecsys® Anti‑SARS‑CoV‑2 S test on positive cases at baseline retested at 6-month.

Higher antibody levels were observed in individuals aged 50–65 years (65.86, 95% CI 22.245; 109.476, p = 0.003), as well as in individuals who had three or more symptoms (40.96, 95% CI 1.7; 80.221, p = 0.041). Moreover, the median antibody titer increased of 11.79 (96% CI 1.33; 22.25, p = 0.03) at 6 the month follow-up (Table 2).

**Table 2:** Multilevel multivariable quantile regression on quantitative antibody levels of positive cases at baseline retested at 6 months.

		Coefficient (95%CI)	p
**Age class**	< = 50		
	> 65	51.72 (− 15.15; 118.60)	p = 0.13
	50–65	65.86 (22.25; 109.48)	p < 0.01
**Sex**	Female		
	Male	15.08 (− 22.72; 52.88)	p = 0.43
**Type of occupation**	Administrative Personnel		
	Physician	− 35.88 (− 96.72; 24.95)	p = 0.24
	Manager	30.23 (− 23.82; 84.29)	p = 0.27
	Retired Individual	− 10.53 (− 79.99; 58.92)	p = 0.76
	Researcher/student	− 36.17 (− 81.37; 9.04)	p = 0.11
	Nurse	− 43.46 (− 87.51; 0.58)	p = 0.05
	Other	− 58.05 (− 107.10; − 8.99)	P = 0.02
**Covid-19 close contact**	No		
	Yes	6.84 (− 26.54; 40.23)	p = 0.69
**No of symptoms**	None		
	One	18.05 (− 21.88; 57.97)	p = 0.37
	Two	30.25 (− 15.61; 76.12)	p = 0.19
	Three or more	40.96 (1.7; 80.22)	p = 0.04
**Smoking**	No		
	Yes	− 49.47 (− 95.48; − 3.47)	p = 0.04
**Comorbidities**	No		
	One	− 8.06 (− 44.26; 28.14)	p = 0.66
	Two or more	45.08 (− 5.87; 96.04)	p = 0.08
**Time follow-up**	Baseline		
	6-month	11.79 (1.33; 22.26)	p = 0.03

Value (95% CI, p-value) represents mean change (95%CI, p-value) in the median of antibody levels associated to each covariate, adjusted for all the others.

## Discussion

In this study we detected a quite different seroconversion rate against SARS-CoV-2 among the five considered groups, being higher in subjects who were diagnosed with a SARS-CoV-2 infection with a previously positive RT-PCR virus test on nasopharyngeal or oropharyngeal swab. Interestingly, the serological test yielded positive results also in 61% of hospitalized patients with a clinical diagnosis of COVID-19 disease based on suggestive clinical and laboratory findings but tested negative on two consecutive naso- or oropharyngeal swabs performed within one week from the hospital admission.

We also showed that humoral immunity against SARS-CoV-2 is maintanined at least for 6 months and we identified factors associated with seroconversion.

The present findings confirm that a population-based serological testing may provide a more precise estimate of the rate of infection among individuals who were not diagnosed by the RT-PCR test, and is of particular relevance for detecting asymptomatic and pauci-symptomatic subjects^10^. In our series, the subjects included in group 3 who have had contact or exposure with confirmed cases of SARS-CoV-2 infection could be comparable to asymptomatic individuals. In these subjects the seroconversion rate was 40%, a figure that, with all the limitations related to the limited number of subjects considered, is aligned with the literature data^10^.

While serological tests are potentially simple and effective as a screening method, they have shown limitations in the diagnosis of acute infections due to the time required for an adaptive immune response to be acquired^11^. However, detecting SARS-CoV-2 antibodies offers the opportunity to confirm past exposure, which may be of particular interest in the case of asymptomatic or subclinical transmissions. In addition, these epidemiologic studies can help identifying the extent of virus spread in households, communities, and specific settings, which could help guide control measures^4,12^.

A review of 54 studies found that antibody tests carried one week after a patient first developed symptoms detected only 30% of patients who had COVID-19. Accuracy increased in to 72% at two weeks and to 94% in the third week^8^. Our study was initiated when the first wave of the pandemic was already flecting down and the interval from clinical recovery of COVID-19 disease and serological test was exceeding 4 weeks and, consequently, the test performance is comparable to the previously available data.

Studies assessing the epidemiology of SARS-CoV-2 among healthcare workers in different countries reported a wide variation of seropositivity prevalence data that ranged from 1.6 to 31.6%^13^. Such a huge variation was likely dependent on variable working settings, types of exposure, rates of infection transmission in the community, use of personal protection equipments, and methodological differences among the studies. In our study, the prevalence rate among physicians working in non-COVID-19 dedicated wards or consultation rooms was 28%, being not different from those working in COVID-19 dedicated wards. Notably, two Italian studies conducted in health care workers in geographical areas with low incidence of SARS-CoV-2 indicated lower seroconversion rates in the range of 3 to 5%^14,15^.

Our study establish that seropositivity was associated with older age, male sex, previous COVID-19 disease, and contact with SARS-CoV-2 infected individuals. The number of symptoms was linearly increased with the chance of seropositivity while smoking was inversely associated. These findings are in line with previous observations on population-based studies, reporting that occupation as healthcare workers^16^ and the severity of disease were associated with antibody presence, while female sex and smoking were associated with lower antibody levels^17^. The correlation between anti-SARS-CoV-2 antibody titer and disease severity, however, is not always consistent across different studies^18^.

The negative association between history of tobacco smoking and serological positivity should be considered with caution. Plausible biologic mechanisms while smoking might be protective against COVID-19 include an anti-inflammatory effect of nicotine, a blunted immune response among smokers and increased nitric oxide in the respiratory tract in addition to the a potential protective role of squamous cell metaplasia, which is commonly associated with tobacco smoking. As of now, however, the claims of a protective effect must be viewed with extreme caution and some studies suggests have the smoking effect should not be interpreted causally given the presence of factors that are likely to have a mediation influence^19,20^.

A relevant question concerns if seropositive results as obtained with the available IgM/IgG immunoassays for SARS-CoV-2 indicate the presence of neutralizing antibodies. In a previously published study, neutralizing antibodies rose in tandem with immunoglobulin titers following symptom onset^21^, and the findings were confirmed in further studies^22,23^. One of these studies showed that a large proportion of convalescent plasma samples have modest antibody levels and that commercially available tests have varying degrees of accuracy in predicting neutralizing antibody activity^23^. Overall, these findings provide immediate clinical relevance to serology results that can be equated to neutralizing antibody activity and could serve as a valuable 'roadmap' to guide the choice and interpretation of serological tests for SARS-CoV-2. However, in a Chinese study neutralizing antibody titers to SARS-CoV-2 appeared to vary substantially and in 117 patients available for a two-week follow-up appointment neutralizing antibody levels had decreased significantly compared to the levels at discharge^24^. More recently, a prospective, longitudinal serological investigation of a large cohort of healthcare workers found the presence of anti-spike antibodies associated with a minimal risk of further SARS-CoV-2 infection in the following 31 weeks of surveillance^25^. These laboratory observations need long-term serial testing to confirm or deny if long term immunity against SARS-CoV-2 confer protection from re-infection.

Our data indicate that at the 6-month follow up the level of humoral immunity was maintained and in some groups the median SARS-CoV-2 antibody level was even increased. Our findings extend current knowledge on whether the immune response to SARS-CoV-2 infection wanes with time. Studies have found that anti-SARS-CoV-2 antibody levels remain stable for several months after infection but there are also reports at variance, suggesting that the antibody titer is rapidly waning, particularly in individuals with milder disease^26^. The rate of decay seem to be dependent on the antigen type, and the anti-S antibodies are reported to be stable for at least 3 months^26^. In a study of > 30,000 individuals with anti-SARS-CoV-2 antibody, neutralization titers correlated with anti-spike binding titers and most individuals who have recovered from mild disease showed moderate to high titers. A longitudinal assessment was performed in a subset of 121 who were tested at different time points observing stable levels for at least 3 months followed by a limited reduction at 5 months^27^. In another study, samples were collected at multiple time points after severe COVID and only 3 out of 70 individuals showed seroreversion of anti-RBD IgG antibodies at 3 months^28^. In a surveillance study on > 3000 healthcare workers who were followed for a median of 4 months, 16% of them tested positive for IgG antibodies. While a fall in titer was seen for anti-nucleocapsid antibodies at 3 months, 94% of the individual with anti-spike antibodies maintained a detectable titer at 6 months, although a quantitative analysis was not done^25^. Also Thangaraj et al.^30^ observed a clear waning of anti-nucleocapside and anti-spike antibodies, but the persistence of neutralizing, anti-receptor binding domain antibodies in 86.2% of participants after more than 6 months from diagnosis and Epaulard et al.^30^ confirmed this finding with a longer follow-up. In their report, neutralizing, anti-receptor binding domain antibodies remain detectable 7 months after infection in 65 of 67 patients, and antibody levels remain stable between 6 months and 1 year.

This type of knowledge about specific serological test and long-term serial blood tests will enable scientists to better understand how long these antibodies persist and if they determine protection against both reinfection and transmission. This information can also help public health officials understand how widespread the outbreak is and could help support the development of treatments and vaccines for COVID-19. In times of limited availability of vaccine doses, the assessment of seropositivity to SARS-CoV-2 in different samples of the population may help in defining a scale of priority for access to vaccination that may be deferred in subjects with elevated antibody levels. Moreover, assessing antibody response to SARS-CoV-2 may help to support a reliable disease-surveillance system and grading community risk.

Strenghts of the study are the contemporary assessment of different groups of individuals living in the same area and the same working place (a university hospital) during the pandemic, thus giving a comprehensive appraisal of the spreading of SARS-CoV-2 infection through individuals with a spectrum of occupational and environmental risk, and its prospective assessment of humoral immunity. Limitations of the study include the limited sample size, the fact that PCR testing at 3 month was done in only 88% of individuals enrolled and that serologic assessment at six months was done only in individuals who were positive at 3 months. Additionally, recall and social desirability biases in reporting symptoms cannot be excluded.

In conclusion, the present study shows that humoral immunity against SARS-CoV-2 virus is not transient; conversely, it may last for several months. Age, sex, contact with infected subjects, history of disease, its clinical severity, and smoking are among the most relevant factors influencing antibody titer.

## Supplementary Information

Supplementary Information.
