# Supplementary material for: Humoral immune response to SARS-CoV-2 in five different groups of individuals at different environmental and professional risk of infection
Source: Sci Rep. 2021 Dec 30;11:24503. doi: 10.1038/s41598-021-04279-4 (PMC8718534; doi:10.1038/s41598-021-04279-4)
Supplement: Supplementary file 1 — Supplementary Information. [file 41598_2021_4279_MOESM1_ESM.pdf]

Humoral immune response to SARS-CoV-2 in five different groups of individuals at different environmental and professional risk of infection.

Silvia Novello[1]*, Massimo Terzolo[2]*, Berchialla Paola[2], Martina Gianetta[1] , Valentina Bianco[1] Francesca Arizio[1], Dalila Brero[2], Anna Maria Elena Perini[2], Adriana Boccuzzi [3], Valeria Caramello[3], Alberto Perboni [4] ,Fabio Bellavia[4], Giorgio Vittorio Scagliotti[1]

Table S1 – Comorbidities, smoking status, and COVID-19 related symptoms in the study groups.

**GROUP**	**1**	**2**	**3**	**4**	**5**	**Overall**	p **value**
N. individuals	(N=120)	(N=89)	(N=128)	(N=246)	(N=406)	(N=989)	
Comorbidities -n (%)							*P<0.01*[2]
None	70 (58)	47 (53)	87 (69)	179 (73)	269 (66)	652 (66)	
One	28 (23)	35 (28)	23(18)	52(21)	80(20)	208(21)	
Two or more	22(19)	17(19)	16(13)	15(6)	57(14)	127(13)	
Smoker	5 (4)	12 (13)	32 (25)	36 (15)	70 (17)	155 (16)	*P<0.01*[2]
COVID-19 related symptoms – N (%)							
None	5 (4)	4 (4.5)	63 (49)	131 (53)	278 (68.5)	481 (49)	*P<0.01*[2]
Cough	70 (58)	43 (48)	19 (15)	41 (16.5)	58 (14)	231 (23)	*P<0.01*[2]
Dyspnea	53 (44)	34 (38)	4 (3)	9 (3.5)	5 (1)	105 (10.5)	*P<0.01*[2]
Asthenia/Muscular aches	75 (62.5)	44 (49.5)	28 (22)	51 (20.5)	42 (10.5)	240 (24.5)	*P<0.01*[2]
Diarrhea	43 (36)	19 (21.5)	19 (15)	30 (12)	27 (6.5)	138 (14)	*P<0.01*[2]
Nausea	28 (23.5)	10 (11)	8 (6)	11 (4.5)	14 (3.5)	71 (7)	*P<0.01*[2]
Conjunctivitis	10 (8.5)	6 (7)	3 (2.5)	10 (4)	13 (3)	42 (4.5)	*P=0.07*[2]
Headache	40 (33.5)	27 (30.5)	14 (11)	61 (25)	38 (9.5)	180 (18)	*P<0.01*[2]
Anosmia	68 (57)	34 (38)	21 (16.5)	25 (10)	14 (3.5)	162 (16.5)	*P<0.01*[2]
Ageusia as first symptom/ total with ageusia	27/79 (34)	16/56 (28.5)	9/46 (19.5)	11/86 (12.8)	7/121 (5.8)	70/388 (18)	*P<0.01*[2]
Other	21 (17.5)	10 (11)	6 (4.7)	13 (5.3)	18 (4.4)	68(6.9)	*P<0.01*[2]
N. of symptoms							*P<0.01*[2]
0	13 (10.8)	9 (10.1)	69 (53.9)	137 (55.7)	282 (69.5)	510 (51.6)	
1	21 (17.5)	32 (36)	33 (25.8)	43 (17.5)	73 (18)	202 (20.4)	
2	27 (22.5)	15 (16.8)	14 (10.9)	34 (13.8)	28 (6.9)	118 (11.9)	
3 or more	59 (49.2)	33 (37.1)	12 (9.4)	32 (13)	23 (5.7)	159 (16.1)	

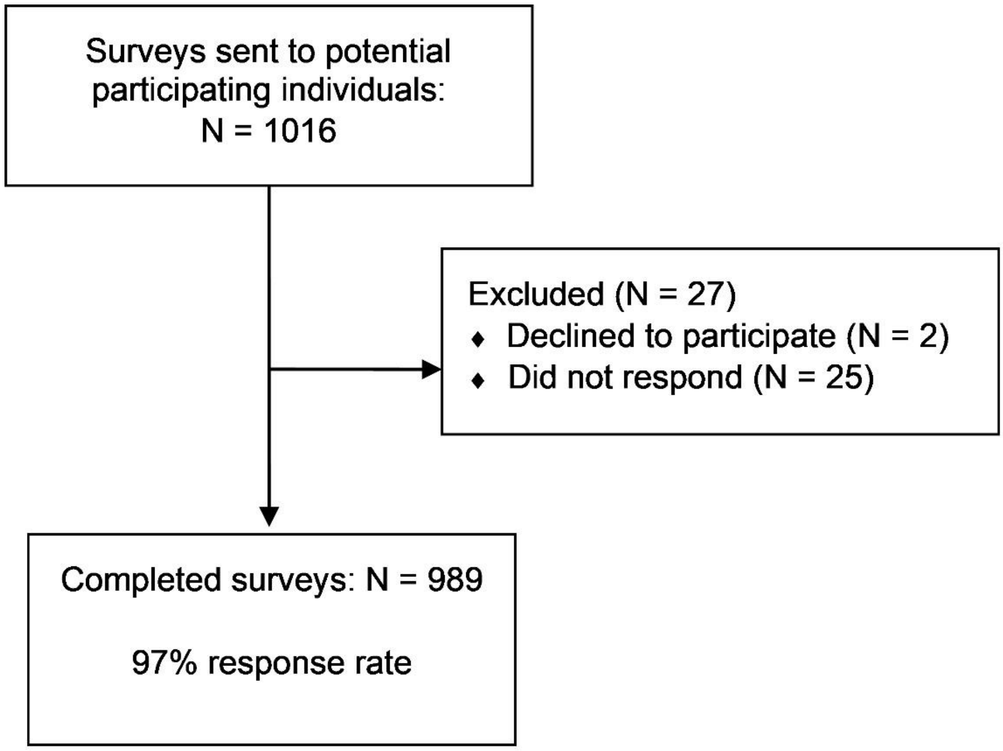

Figure S1. Flow chart of survey study participants
